# Supplementary material for: Hybrid Models and Biological Model Reduction with PyDSTool
Source: PLoS Comput Biol. 2012 Aug 9;8(8):e1002628. doi: 10.1371/journal.pcbi.1002628 (PMC3415397; doi:10.1371/journal.pcbi.1002628)
Supplement: Text S4 — Complete source code for the PyDSTool package (version 0.88.120504). Includes API documentation and help files linking to web pages. This file is identical to the current public release on Sourceforge.net. (ZIP) [file pcbi.1002628.s004.zip › PyDSTool/html/toc-PyDSTool.PyCont.BifPoint-module.html]

xml version="1.0" encoding="ascii"?


BifPoint


# Module BifPoint

---

## Classes

BPoint  
 BTPoint  
 BifPoint  
 BranchPoint  
 CPPoint  
 DHPoint  
 FoldPoint  
 GHPoint  
 HopfPoint  
 LPCPoint  
 NSPoint  
 PDPoint  
 SPoint  
 ZHPoint  

## Variables

\_classes

---

[hide private]
